# The "Quarterly Review" on Hospitals

**Published:** 1893-11-25

**Authors:** 


### The Hospital

An Institutional Journal of

Science, Medicine, Hygiene, Nursing, and News.

*For Hospitals, Asylums, Infirmaries, Dispensaries, Medical Practitioners, Students, and Nurses.*

Vol. XV.—No. 374.] [Nov. 25, 1893.

### The "Quarterly Review" on Hospitals.

The October number of the *Quarterly Review* reminds the public in a long, but by no means exhaustive, article of the vast amount of information and reasoned conclusion which has been accumulated concerning the hospital systems of Great Britain and the world during the past quarter of a century, and puts us upon the inquiry whether or not all this information and those reasoned conclusions, are to be made to render that service to the hospital world which they are capable of rendering. Hospital managers and hospital officials do not belong to the *profanum vulgus*, who can only be made to understand and to act upon large and sound economic principles by whip and spur. On the contrary, they belong, or ought to belong, to the class of educated specialists with whom available knowledge rapidly developes itself into practical progress. By dint of great labour, extending over a long series of years, and pursued in the presence of obstacles of almost every conceivable and inconceivable kind, the lore of the hospital system from the earliest historic times has been gathered from all parts of the world, and brought within a compass which renders it universally available. We desire now to see that learning and these principles put into concrete form in every hospital which has not already placed itself fully abreast of the best knowledge and progress of our own period.

It is always advantageous for us "to see ourselves as others see us," and when "others," as in the present case, are represented by so sober and weighty an authority as the *Quarterly Review*, the advantages are increased mightily. One of the most ardently discussed of all the problems of the hour is the voluntary principle as applied to the maintenance of hospitals. Should hospitals depend for their support upon the voluntary gifts of the public, or should they be maintained at the expense of the rates? On this point the *Quarterly* speaks with no uncertain sound. "When, in his reforming zeal, Henry VIII. altered the constitution of the hospitals," says the writer, "although he confiscated for his own use a large share of their endowments, he really helped to place them on a permanently healthy basis; for by this simple process of confiscation, though modified in part by some small re-endowments, he established the modern hospital—the hospital supported by voluntary contributions. The voluntary contribution gives a right of investigation to the contributor." This theoretical contention of the *Quarterly Review* is fully borne out by the facts of long experience. In no single instance, so far as we are aware, has it ever been known that a rate-supported hospital has provided for the sick so scientific, so sympathetic, and so practically serviceable a home as has been provided in every considerable town by the voluntary gifts of the charitable. Whatever may prove to be the case in the future, it is certain that at the present time the best of the "Voluntary Hospitals," as they are called, are about as perfect in their administration and efficiency as any merely human institutions can be, and beyond comparison superior to any that are supported by the rates.

The writer in the *Quarterly* places his finger upon a blot which is not altogether creditable to the voluntary system. "The management of a hospital," says he, "falls practically into the hands of a very few governors, who constitute, formally, or informally, a weekly or monthly board, and it is to these that the officials make their reports; it is they who criticise or suggest, who approve or condemn new methods, who point out the necessity for changes and improvements, while the sleeping governors, as they may be called, give a formal and unthinking sanction to the recommendation of the smaller body. Occasionally, indeed, they refuse to do so; and it is a grievance of those who take an active interest in an institution that they are liable to be swamped and over-ridden at any time by the great body of the governors." This is undoubtedly one of the weak places in the armour of voluntaryism at hospitals. But it is a weakness which is now very clearly perceived. Perhaps it was not within the province of the Quarterly Reviewer to suggest some practicable modification of the present system. But it is certainly not only within the province, but within the imperative duty of enlightened hospital politicians to set about the general establishment of new and more approved principles of administration. It seems to us quite absurd to give the power of veto to the whole body of governors when not one-twentieth part of those exercising the veto knows really anything about the matter in hand. It is like making the whole body of practising barristers a legal court of appeal in a difficult case, instead of two or three of the most distinguished judges in the land. We venture to suggest that the true function of a body of governors is not to vote at all in administrative affairs, but to select from among their number a chosen few who have time, mental capacity, and willingness to act as cabinet ministers whilst delegating the details of administration to subordinate committees. This small consultative body should be renewed from among the mass of governors periodically, and should speak and act with the full authority of the whole body of the governors. A reform so apparently simple as this, if carried out in the practical spirit of the modern politician, would go far to effect a most advantageous revolution in the administration of the whole circuit of hospital affairs.

On another very vexed question in our hospital system the writer in the *Quarterly* lays an unsparingly critical hand. He might be a general practitioner of medicine, though we happen to know that he is not, so ardently does he espouse the cause of those practitioners, who, rightly or wrongly, think they are greviously injured by the unequal competition of hospitals. "It is certain," says he, "that doctors do not thrive where hospitals abound, and it seems evident that if all who receive free medical relief are hopelessly unable to pay for it, there are districts, at least in the metropolis, which must be on the verge of general bankruptcy. Take the evidence of Lieut.-Colonel Montefiore, with regard to the district of Marylebone, which cannot be regarded as one of the poorest districts in London." This is a portion of the evidence referred to, and it was given before the Select Committee of the House of Lords so recently as 1890. Said Colonel Montefiore, "The parish of Marylebone has an area of 1,506 acres and a population of 155,004. . . . We find that in 1885 the following numbers received gratuitous treatment—64,516 from six free hospitals, 7,567 from three free dispensaries, and 7,731 from the Poor Law, making a total of 79,814," or some thousands more than half of the whole population of Marylebone. This fact, standing as it does among large numbers of similar facts, seems to have astounded the writer in the *Quarterly*, as well it might. It is so altogether startling and intolerable that we ourselves prefer to say nothing at all about it, lest we should be tempted to say more than we ought, if that were possible.

Ought Pay Hospitals to be encouraged? The *Quarterly Review* answers this question, on the whole, affirmatively. But then it insists that they shall be real Pay Hospitals, not make-believes. That is to say those persons who, being able to pay private practitioners, nevertheless prefer to be treated at hospitals, shall pay at least as much to the hospital for medical care, nursing, and food, as they would be obliged to pay if treated in their own homes. In other words the Pay Hospital shall not use the charitable contributions of the benevolent for the purpose of underselling the private practitioner. This, it seems to us is the rational solution of the difficulty. But the writer, who appears to be particularly well informed, tells us that this solution cannot be applied, because of the determined resistance of the Honorary Medical Staff of the hospitals. Those who are within the inner circle of hospital politics know that the dead-lock thus pointed out by the *Quarterly* is the true and veritable secret of the whole mystery. For our own part we do not think that the difficulty will prove to be permanently insurmountable. The honorary physicians and surgeons of hospitals, though not men of affairs, are nevertheless persons of great natural intelligence and deep, if somewhat narrow, culture. Undoubtedly they will in no long time perceive that the present chaos and confusion worse confounded is as derogatory to their own dignity as it is the source of immeasurable heart-burnings among the practitioners of medicine, and the despair of the ever increasing number of hospital managers, who are men of wide practical experience in general affairs.

We have endeavoured to draw attention to some of the most important points in a singularly able and temperate article. It is our hope that hospital physicians and surgeons, managers, and the thoroughly business-like class of secretaries, matrons, and other superior officials, will "read, mark, learn, and inwardly digest" this peculiarly opportune "Tract for the Times."

